# Impact of COVID-19 versus other pneumonia on in-hospital mortality and functional decline among Japanese dialysis patients: a retrospective cohort study

**DOI:** 10.1038/s41598-024-55697-z

**Published:** 2024-03-02

**Authors:** Ken Ikenouchi, Daiei Takahashi, Shintaro Mandai, Mizuki Watada, Sayumi Koyama, Motoki Hoshino, Naohiro Takahashi, Wakana Shoda, Tamaki Kuyama, Yutaro Mori, Fumiaki Ando, Koichiro Susa, Takayasu Mori, Soichiro Iimori, Shotaro Naito, Eisei Sohara, Kiyohide Fushimi, Shinichi Uchida

**Affiliations:** 1 Department of Nephrology, Graduate School of Medical and Dental Sciences, Tokyo Medical and Dental University, 1-5-45 Yushima, Bunkyo, Tokyo, 113-8519 Japan; 2 Department of Nephrology, Musashino Red Cross Hospital, 1-26-1, Kyonann-cho, Musashino-shi, Tokyo, 180-8610 Japan; 3 Department of Health Policy and Informatics, Graduate School of Medical and Dental Sciences, Tokyo Medical and Dental University, 1-5-45 Yushima, Bunkyo, Tokyo, 113-8519 Japan

**Keywords:** Nephrology, Health care, End-stage renal disease, Viral infection

## Abstract

Coronavirus disease 2019 (COVID-19) affects both life and health. However, the differentiation from other types of pneumonia and effect of kidney disease remains uncertain. This retrospective observational study investigated the risk of in-hospital death and functional decline in ≥ 20% of Barthel Index scores after COVID-19 compared to other forms of pneumonia among Japanese adults, both with and without end-stage kidney disease (ESKD). The study enrolled 123,378 patients aged 18 years and older from a national inpatient administrative claims database in Japan that covers the first three waves of the COVID-19 pandemic in 2020. After a 1:1:1:1 propensity score matching into non-COVID-19/non-dialysis, COVID-19/non-dialysis, non-COVID-19/dialysis, and COVID-19/dialysis groups, 2136 adults were included in the analyses. The multivariable logistic regression analyses revealed greater odds ratios (ORs) of death [5.92 (95% CI 3.62–9.96)] and functional decline [1.93 (95% CI 1.26–2.99)] only in the COVID-19/dialysis group versus the non-COVID-19/non-dialysis group. The COVID-19/dialysis group had a higher risk of death directly due to pneumonia (OR 6.02, 95% CI 3.50–10.8) or death due to other diseases (OR 3.00, 95% CI 1.11–8.48; versus the non-COVID-19/non-dialysis group). COVID-19 displayed a greater impact on physical function than other types of pneumonia particularly in ESKD.

## Introduction

Coronavirus disease 2019 (COVID-19) is a highly infectious respiratory disease caused by the severe acute respiratory syndrome coronavirus 2. It affects the respiratory and psychological performance of patients^1,2^. The emergence and pandemic of COVID-19 have substantially altered conventional healthcare systems, including isolation protocols for infectious or febrile patients and universal precautions^3–5^. Accumulating evidence suggests that COVID-19 is directly and indirectly associated with a higher risk of decline in activities of daily living (ADL). Even after recovering from the acute phase, COVID-19 survivors experience a decline in physical function, ADL, and health-related quality of life, with recovery taking several months^6^. However, it is not fully understood whether COVID-19 specifically affects physical function compared to non-COVID-19 pneumonia.

Chronic kidney disease (CKD) affects over 700 million people worldwide and represents a major public health burden^7,8^. CKD not only affects the filtration of uremic solutes or toxins but also impacts multiple organs, such as cardiovascular diseases, glucose intolerance, and musculoskeletal disorders^9–12^. A previous study reported that 73% of patients with CKD initiating chronic dialysis already suffer from frailty^13^. Patients with CKD are particularly vulnerable to infections and are at an increased risk of experiencing severe symptoms in relation to various infectious diseases. It is known that the risk of in-hospital mortality due to pneumonia is 4.1- to 23.4-fold higher in patients with CKD, particularly in those on dialysis^14^. A Western report also demonstrated that the adjusted risk of mortality in patients with end-stage kidney disease (ESKD) hospitalized with COVID-19 in 2020 was 37% higher than in COVID-19 patients without ESKD^15^. Not only mortality but also the risk of decline in ADL is expected to be even higher in frail patients on dialysis. However, there is limited research on the risk of decline in ADL among dialysis patients with COVID-19. Furthermore, the impact of ESKD on ADL outcomes after hospitalization with COVID-19 is yet to be determined.

To address these issues, this study was designed to investigate the risk of in-hospital death and decline in ADL after COVID-19 compared to non-COVID-19 pneumonia in patients on chronic dialysis using an inpatient administrative claims database in Japan. Our hypothesis is that patients with COVID-19 who are on chronic dialysis (COVID-19/D) have a higher risk of in-hospital mortality and loss of ADL than the other three groups (non-COVID-19/non-dialysis [non-COVID-19/ND], COVID-19/non-dialysis [COVID-19/ND], non-COVID-19/dialysis [non-COVID-19/D]), due to underlying complex comorbidities and frailty. To compare the outcomes, we matched the patients into these four groups.

## Methods

### Source of data

The study participants were identified from the Diagnosis Procedure Combination (DPC) inpatient database, which is an administrative claims database in Japan. This database includes more than 1000 hospitals, including all 82 university hospitals, and covers more than half of all admissions in the country^16^. The DPC database provides information on various aspects such as diagnosis and comorbidities at hospital admission and cause of death coded according to the International Classification of Disease and Related Health Problems, 10th Revision (ICD-10)^17^. It also includes patient information such as age, gender, Body Mass Index (BMI), admission and discharge status, ADL at admission and discharge, and a comorbidity score known as the Charlson Comorbidity Index^18^, which is updated for risk adjustment^19,20^. This study was performed in accordance with the ethical principles laid down in the 1964 Declaration of Helsinki, and was approved by the ethics committee of Tokyo Medical and Dental University (No. M2000-788). The requirement for informed consent was waived by the ethics committee of Tokyo Medical and Dental University due to the anonymous nature of the data.

In 2020, there were 338,256 pneumonia-related cases as any of a primary diagnosis during hospitalization, reason for admission, or disease that required the highest medical care cost in the DPC database. The inclusion criteria for this study were patients who were at least 18 years old, had a hospital stay of at least 24 h, and had either COVID-19 or pneumonia as the primary diagnosis for hospitalization (Fig. S1). COVID-19 and non-COVID-19 pneumonia were recognized with the ICD-10 codes. Several exclusion criteria were applied, including second or subsequent admissions, death within 24 h of admission, a BMI less than 15 or greater than 50, incomplete information on BMI, ADL, and admission type (emergent or non-emergent), patients with aspiration pneumonia, and patients who initiated hemodialysis or peritoneal dialysis during hospitalization. A total of 123,378 patients were included before matching, comprising 66,692 non-COVID-19/ND patients, 54,132 COVID-19/ND patients, 1894 non-COVID-19/D patients, and 660 COVID-19/D patients. After a propensity score matching (PSM), 2136 patients were divided into four subgroups, each consisting of 534 patients (Fig. S1).

Patients who received maintenance hemodialysis and peritoneal dialysis were recognized with the coding of patient care procedures as follows: chronic maintenance hemodialysis with < 4 h per session, ≥ 4 h and < 5 h per session, ≥ 5 h per session, or chronic maintenance hemodiafiltration or continuous peritoneal dialysis. One COVID-19/D patient was dependent of both hemodialysis and peritoneal dialysis and the remaining dialysis patients were on maintenance hemodialysis. There were 13 patients post transplantation, including 7 post kidney transplant, 5 post stem cell transplant, and 1 post liver transplant patients.

### Patient characteristics

The Barthel Index scores at admission and discharge were calculated based on 10 functional abilities: feeding, bathing, dressing, grooming, toileting, bowel control, bladder control, chair transfer, ambulation, and climbing stairs^21^. The Barthel Index ranges from 0 to 100 points, with the highest scores indicating greater independence in physical functions and lower scores indicating a more bedridden status. The Barthel Index scores were used to classify the level of dependence into four groups: total dependence (0–20 points), severe dependence (21–60 points), moderate dependence (61–90 points), and mild dependence or complete independence (91–100 points)^22,23^. In addition, other clinical data on inpatients were collected, including age, gender, BMI, dialysis dependency, the updated Charlson Comorbidity Index excluding renal disease^20,23^, comorbidities, and admission type (emergency or non-emergency). The age groups used in the analysis were 18–49, 50–59, 60–69, 70–79, and greater than 80 years^24^.

### Outcomes

The primary outcome of this study was the occurrence of in-hospital deaths from any cause. The secondary outcome was a decline in physical function, defined as a decrease of at least 20% in the Barthel Index score at discharge compared to that at admission. We also evaluated a risk of death directly from pneumonia or death due to other diseases. The database identified deaths from COVID-19 or non-COVID-19 pneumonia as a primary diagnosis during hospitalization or those from other reasons. Other outcomes included hospital length of stay and medical care cost. The long-term hospitalization was defined as a stay of 30 days or longer. The high medical cost was defined as the highest quartile of participants. Patients were followed until discharge, transfer, or in-hospital death^20^.

### Data analyses

Baseline characteristics were presented as numerical values (%) or medians (interquartile ranges). The Wald confidence interval for proportions was examined. We estimated the propensity score using a logistic regression model. To minimize potential confounding effects, differences in age (18–49, 50–59, 60–69, 70–79, and greater than 80 years) and sex were adjusted. Based on the propensity score using a 1:1 scheme, Non-COVID-19/D patients (prematching, *N* = 1894; postmatching, *N* = 534) were first matched with COVID-19/D patients (prematching, *N* = 660; postmatching, *N* = 534). Subsequently, COVID-19/ND (prematching, *N* = 54,132; postmatching, *N* = 534) and Non-COVID-19/ND patients (prematching, *N* = 66,692; postmatching, *N* = 534) were adjusted with each COVID-19/D patient. The caliper width used for matching was set at 0.25 of the standard deviation of the propensity score. The cumulative hazard after hospitalization for pneumonia was assessed using the Nelson–Aalen estimator among the four groups. The log-rank test was used for a statistical comparison. Risks of mortality or functional decline were estimated using logistic regression analyses among postmatching patients, adjusting for age, gender, BMI, Barthel Index score at admission, and Charlson Comorbidity Index score. Statistical analyses were performed using JMP Pro 12.0 software (SAS Institute Inc., Cary, USA). *p*-values of less than 0.05 were considered statistically significant.

## Results

### Patient characteristics

The characteristics of the dialysis and non-dialysis patients hospitalized with COVID-19 or non-COVID-19 pneumonia (*n* = 123,378) in Japan during 2020 are listed in Table S1. Before PSM, the prematching patients had an age ranging from 56 to 80 years, a percentage of women ranging from 22 to 43%, and a BMI ranging from 21 to 23 kg/m^2^. After PSM, a total of 2136 patients were divided into four subgroups, each consisting of 534 patients: non-COVID-19/ND, COVID-19/ND, non-COVID-19/D, and COVID-19/D (Table 1). The median age for each subgroup was 69 years, with the percentage of women ranging from 26 to 27% and a BMI ranging from 21 to 24 kg/m^2^. In comparison to the COVID-19 group, the non-COVID-19 group exhibited lower Barthel Index scores. The patients with non-COVID-19 pneumonia had higher proportions of cardiovascular disease and higher Charlson Comorbidity Index scores than the patients with COVID-19. Furthermore, Charlson Comorbidity Index scores were higher in the dialysis group than in the non-dialysis group.

**Table 1:** Characteristics of patients hospitalized with pneumonia in Japan during 2020.

Variable	Non-COVID-19/ND	COVID-19/ND	Non-COVID-19/D	COVID-19/D	*p-*value
	(*n* = 534)	(*n* = 534)	(*n* = 534)	(*n* = 534)	
Age, years	69 (56–78)	68 (56–77)	69 (57–78)	68 (56–77)	0.3943
18–49	72 (13.48)	72 (13.48)	64 (11.99)	72 (13.48)	1.0000
50–59	101 (18.91)	101 (18.91)	109 (20.41)	101 (18.91)	
60–69	111 (20.79)	111 (20.79)	111 (20.79)	111 (20.79)	
70–79	152 (28.46)	152 (28.46)	152 (28.46)	152 (28.46)	
≥ 80	98 (18.35)	98 (18.35)	98 (18.35)	98 (18.35)	
Female	137 (25.66)	137 (25.66)	144 (26.97)	137 (25.66)	0.9492
Body Mass Index, kg/m^2^	21.30 (18.48–24.43)	23.76 (21.47–26.12)	21.37 (18.74–25.00)	22.96 (20.34–25.79)	< 0.0001
Barthel Index score					
0–20	110 (20.60)	56 (10.49)	121 (22.66)	72 (13.48)	< 0.0001
21–60	71 (13.30)	29 (5.43)	98 (18.35)	66 (12.36)	
61–90	58 (10.86)	22 (4.12)	55 (10.30)	43 (8.05)	
91–100	295 (55.24)	427 (79.96)	260 (48.69)	353 (66.10)	
Charlson comorbidity					
Myocardial infarction	8 (1.50)	7 (1.31)	14 (2.62)	6 (1.12)	0.2121
Congestive heart failure	91 (17.04)	18 (3.37)	188 (35.21)	34 (6.37)	< 0.0001
Peripheral vascular disease	5 (0.94)	7 (1.31)	29 (5.43)	6 (1.12)	< 0.0001
Cerebrovascular disease	39 (7.30)	17 (3.18)	50 (9.36)	23 (4.31)	< 0.0001
Dementia	34 (6.37)	15 (2.81)	17 (3.18)	9 (1.69)	< 0.0001
Chronic pulmonary disease	113 (21.16)	36 (6.74)	33 (6.18)	17 (3.18)	< 0.0001
Rheumatologic disease	22 (4.12)	6 (1.12)	8 (1.50)	4 (0.75)	< 0.0001
Peptic ulcer disease	19 (3.56)	15 (2.81)	16 (3.00)	13 (2.43)	0.7466
Mild liver disease	19 (3.56)	14 (2.62)	11 (2.06)	5 (0.94)	0.0354
Diabetes without chronic complications	88 (16.48)	94 (17.60)	79 (14.79)	51 (9.55)	0.0001
Diabetes with chronic complications	19 (3.56)	10 (1.87)	143 (26.78)	108 (20.22)	< 0.0001
Hemiplegia or paraplegia	0 (0.00)	0 (0.00)	3 (0.56)	1 (0.19)	0.2025
Any malignancy, including leukemia and lymphoma	69 (12.92)	19 (3.56)	47 (8.80)	10 (1.87)	< 0.0001
Moderate or severe liver disease	3 (0.56)	0 (0.00)	1 (0.19)	2 (0.37)	0.5301
Metastatic solid tumor	16 (3.00)	1 (0.19)	1 (0.19)	0 (0.00)	< 0.0001
AIDS/HIV	1 (0.19)	2 (0.37)	0 (0.00)	1 (0.19)	0.9060
Charlson Comorbidity Index					
0	232 (43.45)	430 (80.52)	13 (2.43)	150 (28.09)	< 0.0001
1–2	208 (38.95)	84 (15.73)	283 (53.00)	325 (60.86)	
≧ 3	94 (17.60)	20 (3.75)	238 (44.57)	59 (11.05)	

Data are presented as numbers (percentages) or medians (interquartile ranges). To compare the four groups, one-way analysis of variance was used for continuous variables, while a chi-squared test was used for categorical variables.

*D* dialysis, *non-COVID-19* non-COVID-19 pneumonia.

^a^ND was defined as non-dialysis patients.

### Rates for in-hospital overall death or decline in physical function following pneumonia

**Figure 1:**
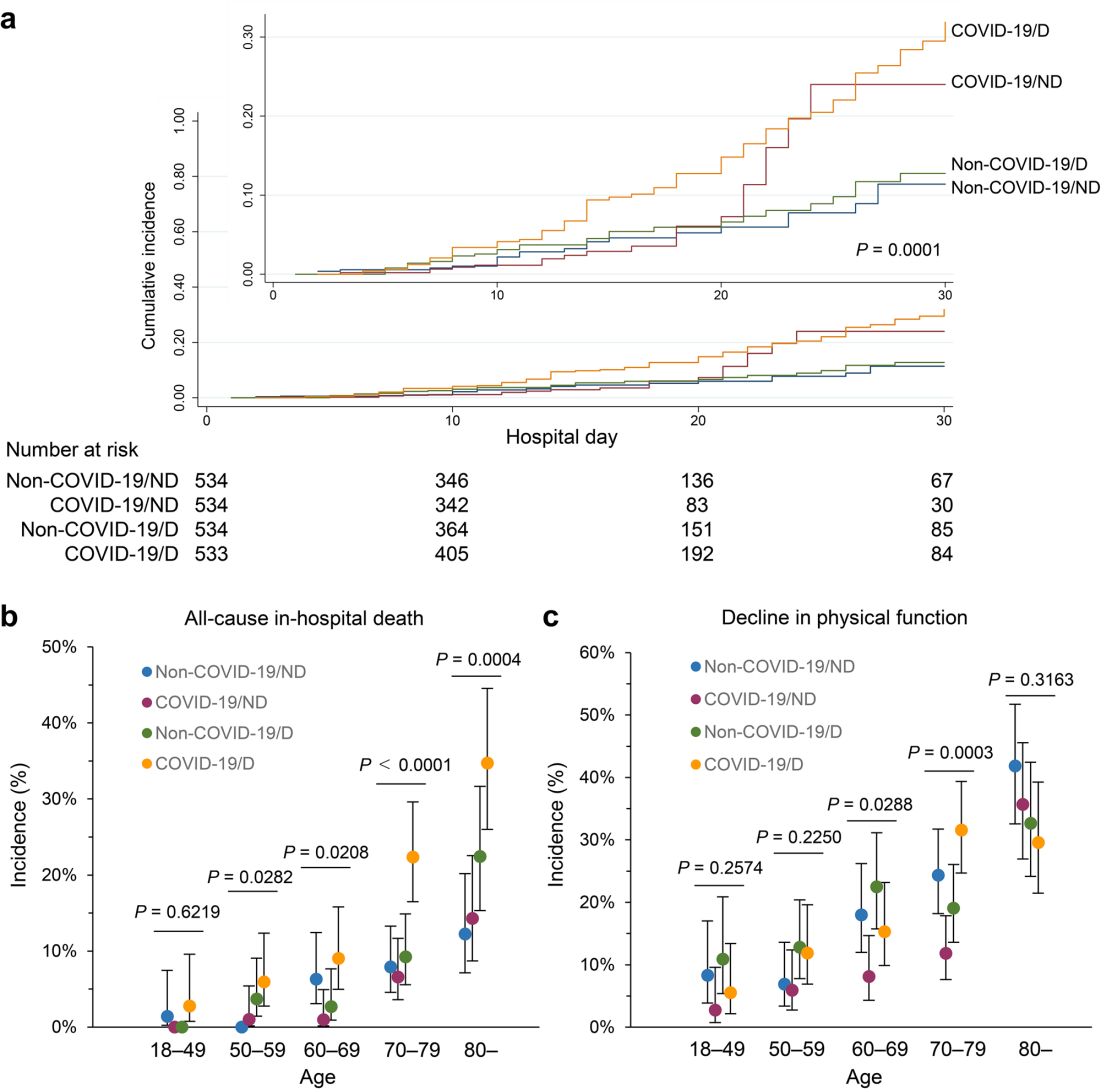
In-hospital death and decline in physical function after coronavirus disease 2019 (COVID-19) and other types of pneumonia in Japan. (**a**) The Nelson–Aalen estimator of the cumulative hazard among the non-COVID-19/non-dialysis, COVID-19/non-dialysis, non-COVID-19/dialysis, and COVID-19/dialysis groups within 30 days after hospitalization. A statistical comparison was performed with the log-rank test (*p* = 0.0001). (**b,c**) Prevalence of overall in-hospital death (**b**) and functional decline after survival (**c**) in each age stratum. Each circle represents a mean, and the solid lines represent the corresponding 95% confidence intervals (CIs) among non-COVID-19/non-dialysis, COVID-19/non-dialysis, non-COVID-19/dialysis, and COVID-19/dialysis groups. A chi-squared test was used to compare the four groups. The decline in physical function was defined as a 20% or more decline in Barthel Index scores at discharge from admission. *CI* confidence interval, *ND* non-dialysis patients, *D* dialysis, *non-COVID-19* non-COVID-19 pneumonia.

In our study cohort, a total of 187 all-cause in-hospital deaths were observed. The mortality rates were 6.0% (95% confidence interval [CI] 4.28–8.34), 4.9% (95% CI 3.34–7.04), 8.1% (95% CI 6.03–10.7), and 16.1% (95% CI 13.2–19.5) in the non-COVID-19/ND, COVID-19/ND, non-COVID-19/D, and COVID-19/D groups, respectively. The Nelson-Aalen estimator, used for survival estimation, revealed that the COVID-19/D group had the highest risk of overall death within 30 days after admission (Fig. 1A). When stratified by age, the mortality rates increased with advancing age in all groups (Fig. 1B). Notably, the COVID-19/D group exhibited particularly high mortality rates among individuals aged 70 years and older.

The percentages of patients who experienced functional decline after survival were 20.8% (95% CI 17.6–24.4), 13.1% (95% CI 10.5–16.2), 20.0% (95% CI 16.9–23.6), and 20.6% (95% CI 17.4–24.2) in the non-COVID-19/ND, COVID-19/ND, non-COVID-19/D, and COVID-19/D groups, respectively (Fig. 1C). With increasing age, the rates of functional decline increased in all groups.

### Risk of in-hospital death and decline in physical function among non-COVID-19/non-dialysis, COVID-19/non-dialysis, non-COVID-19/dialysis, and COVID-19/dialysis patients

**Figure 2:**
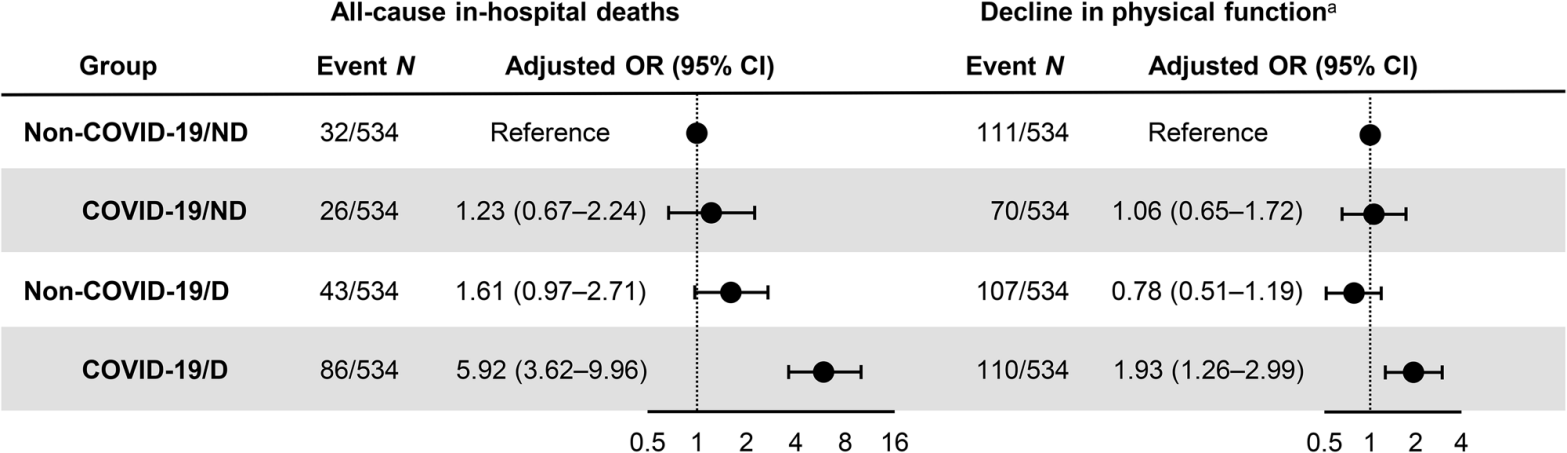
Risk of in-hospital death and decline in physical function among non-coronavirus disease 2019 (COVID-19)/non-dialysis, COVID-19/non-dialysis, non-COVID-19/dialysis, and COVID-19/dialysis patients. Multivariable logistic regression models were adjusted for age, gender, body mass index, Barthel Index score on admission, and Charlson Comorbidity Index score. Each circle represents a point estimate of odds ratios (ORs), and the solid lines represent the corresponding 95% confidence intervals (CIs). ^a^The decline in physical function was defined as a 20% or more decline in Barthel Index scores at discharge from admission. *CI* confidence interval, *OR* odds ratio, *ND* non-dialysis patients, *D* dialysis, *non-COVID-19* non-COVID-19 pneumonia.

To compare the risk of death and decline in physical function among the groups, logistic regression analyses were performed. The results, as shown in Fig. 2, revealed that the COVID-19/ND group had similar odds of death compared to the non-COVID-19/ND group (odds ratio [OR] 1.23, 95% CI 0.67–2.24, *p* = 0.5). The non-COVID-19/D group had 1.61-fold higher odds of death (95% CI 0.97–2.71, *p* = 0.067), while the COVID-19/D group had a significantly higher risk of death (OR 5.92, 95% CI 3.62–9.96, *p* < 0.0001) than the non-COVID-19/ND group.

In terms of functional decline, logistic regression analyses were performed for a minimum 20% decline in Barthel Index scores. The ORs of functional decline were not significantly increased in the COVID-19/ND (OR 1.06, 95% CI 0.65–1.72, *p* = 0.8) and non-COVID-19/D (OR 0.78, 95% CI 0.51–1.19, *p* = 0.3) groups compared to the non-COVID-19/ND group. However, only the COVID-19/D group showed a higher risk of functional decline (OR 1.93, 95% CI 1.26–2.99, *p* = 0.0025) than the non-COVID-19/ND group.

The logistic regression models adjusting for diabetes mellitus instead of Charlson Comorbidity Index showed the similar odds of death and functional decline in the COVID-19/D group (Fig. S2). To further examine the risks of earlier or later deaths, we performed logistic regression analyses for a risk of overall death within 20 days or ≥ 21 days after hospitalization. As a result, the COVID-19/D group was at a greater risk of overall death within 20 days and ≥ 21 days after admission (Fig. S3a).

### Risk of in-hospital death primarily due to pneumonia or other comorbidities among non-COVID-19/non-dialysis, COVID-19/non-dialysis, non-COVID-19/dialysis, and COVID-19/dialysis patients

**Figure 3:**
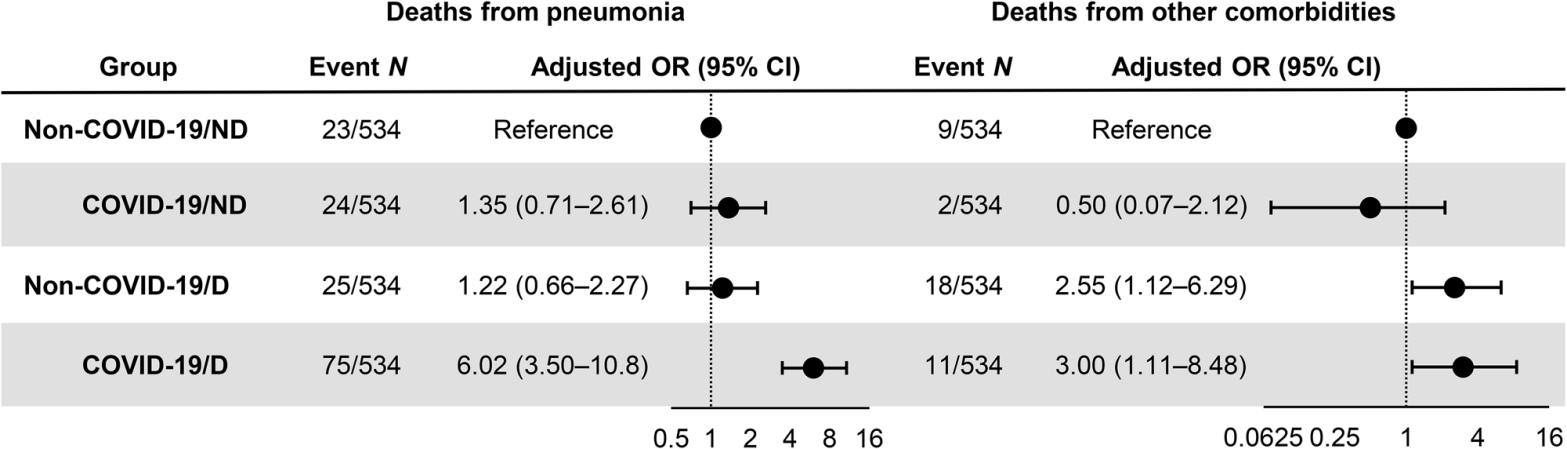
Risk of in-hospital death primarily due to pneumonia or other comorbidities among non-coronavirus disease 2019 (COVID-19)/non-dialysis, COVID-19/non-dialysis, non-COVID-19/dialysis, and COVID-19/dialysis patients. Multivariable logistic regression models were adjusted for age, gender, Body Mass Index, Barthel Index score on admission, and Charlson Comorbidity Index score. Each circle represents a point estimate of odds ratios (ORs), and the solid lines represent the corresponding 95% confidence intervals (CIs). *CI* confidence interval, *OR* odds ratio, *ND* non-dialysis patients, *D* dialysis, *non-COVID-19* non-COVID-19 pneumonia.

To assess whether the causes of death were directly attributed to pneumonia or diseases other than pneumonia, the primary reasons for in-hospital mortality were examined in each group. Figure 3 shows the ORs for direct causes of in-hospital mortality due to pneumonia and other comorbidities. It was found that the non-COVID-19/D and COVID-19/D groups on chronic dialysis were more likely to die due to causes other than pneumonia (OR 2.55; 95% CI 1.12–6.29; OR 3.00, 95% CI 1.11–8.48; *p* = 0.030, respectively) compared to the non-COVID-19/ND group. Notably, the non-COVID-19/D group had a similar risk of death directly due to non-COVID pneumonia (OR 1.22, 95% CI 0.66–2.27), while the COVID-19/D group had a greater risk of death from COVID-19 (OR 6.02, 95% CI 3.50–10.8, *p* < 0.0001). The COVID-19/D group also displayed the highest risk of death from COVID-19 both within 20 days and ≥ 21 days after hospitalization (Fig. S3b). These findings suggest that patients on dialysis are more likely to develop severe pneumonia and subsequent comorbidities under the COVID-19 condition, but not under other types of pneumonia.

To investigate the relationship between hospital stay and outcomes, the length of hospitalization and medical expenses were examined. As shown in Fig. S4, patients on dialysis in the COVID-19/D and non-COVID-19/D groups had a higher risk of long-term hospitalization (OR 1.61, 95% CI 1.11–2.34, *p* < 0.013 and OR 1.37, 95% CI 0.96–1.96, *p* = 0.085, respectively). In contrast, the COVID-19/ND group had a significantly lower risk of long-term hospitalization (OR 0.51, 95% CI 0.32–0.82, *p* < 0.0054). The increased comorbidities and isolation period might contribute to longer hospital stays for patients on dialysis. Logistic regression analyses were also performed to assess the risk of high medical costs, defined as the highest quartile of participants. Notably, the COVID-19/D group was found to be associated with higher medical costs (OR 13.2, 95% CI 9.18–19.1, *p* < 0.0001).

## Discussion

This observational cohort study, using a national sample, aimed to elucidate the risks of in-hospital mortality and functional decline after COVID-19 or other types of pneumonia during the initial three waves of the COVID-19 pandemic. To further compare this association between patients with and without ESKD, PSM was performed within four groups: non-COVID-19/ND, COVID-19/ND, non-COVID-19/D, and COVID-19/D. The findings revealed that the non-COVID-19/D group, and particularly the COVID-19/D group, had a higher risk of in-hospital mortality. The increased mortality in the COVID-19/D group was attributed to deaths due to both COVID-19 and comorbidities other than pneumonia. Despite a shorter hospital stay in the COVID-19/ND group compared to the non-COVID-19/ND group, the risks for functional decline were comparable between the two groups. Notably, the COVID-19/D group demonstrated the highest risks of a hospital stay of 30 days or longer and functional decline compared to the other three groups.

The SARS-CoV-2 variants of concern were B1.1.7 (or alpha) and B.1.351 (or beta) during 2020. The initial Japanese study conducted in 2020 revealed that the crude mortality rate attributed to COVID-19 in patients on dialysis was 20 times higher than that in the general population^25^. The findings were consistent with reports from the United States and Europe, which also reported an increased risk of death from COVID-19 in patients on dialysis compared to non-dialysis patients^14,26^. The present study further demonstrated that dialysis patients with non-COVID-19 and COVID-19 pneumonia exhibited a higher risk of mortality than non-dialysis patients. These findings align with previous studies reporting that patients on dialysis are immunocompromised^8,27^. Notably, the mortality risk associated with COVID-19 was much greater than that for other types of pneumonia in patients with ESKD. Regarding the direct causes of death, the mortality risks due to diseases other than pneumonia were similarly higher in both the COVID-19/D and non-COVID-19/D groups than in the ND groups. This finding is consistent with previous studies highlighting the complex nature of disease and the presence of multiple comorbidities in patients with ESKD^28^. It is noteworthy that the risk of death directly attributed to pneumonia was not increased in the non-COVID-19/D group, while it was markedly higher in the COVID-19/D group. These findings suggest that COVID-19 leads to more severe pneumonia and comorbidities in patients with kidney diseases compared to other types of pneumonia.

In the present study, an increased risk of decline in ADL was observed in dialysis patients with COVID-19, with approximately twice the odds compared to other groups. This could be attributed to the longer length of hospital stay, which generally leads to a decline in ADL^29^. However, it is worth noting that the increase in the length of hospital stay was also marginally significant for dialysis patients with non-COVID-19 pneumonia. Furthermore, despite a much shorter hospital stay in the COVID-19/ND group, the risk of ADL decline was equally high compared to the non-COVID-19/ND group. These findings suggest that COVID-19 specifically affects the musculoskeletal system and physical function during hospitalization more than non-COVID-19 pneumonia.

It has been reported that COVID-19 leads to a decline in ADL^30^. However, the difference in the impact of COVID-19 compared to other types of pneumonia on physical function has not been fully understood. This study is the first to compare this using a nationwide survey with the PSM of the groups. The potential mechanisms for the impact of COVID-19 on physical function include the cytokine storm (e.g., tumor necrosis factor-alpha and interleukin 6) induced by the virus, which may directly cause a decrease in muscle mass and strength by decreasing the synthesis and increasing the degradation of muscle proteins^31,32^. Other factors that may contribute to this decline include myotoxic drugs like steroids and Intensive Care Unit admissions^30^. However, further studies are needed to elucidate the remaining factors and mechanisms in order to develop specific interventions to mitigate the loss of muscle mass, strength, and physical function during COVID-19.

It is possible that the isolation of patients with COVID-19 contributed to the decline in ADL in patients on dialysis. During the earlier waves of the COVID-19 pandemic, early discharge and home recuperation were usually recommended until sufficient evidence was accumulated. In contrast, dialysis patients with COVID-19 presumably required a longer hospital stay because of a need for a hospital-based isolation during dialysis sessions. Due to their underlying frailty, the combination of isolation and the direct impact of COVID-19 pneumonia may have a substantially critical effect on patients on dialysis compared to those with normal kidney function. Early rehabilitation intervention after COVID-19 has been reported to help maintain ADL (17). Currently, there are no guidelines or consensus on early intervention in the acute phase of COVID-19. Our findings may have clinical implications for the isolation protocols of patients with COVID-19 and the implementation of universal precautions, particularly for patients with underlying kidney disease.

The strength of this study lies in its well-validated, large-scale design, which provided a nationally representative sample of the Japanese population. However, there are several limitations that should be acknowledged. First, our study specifically focused on the initial three waves of the COVID-19 epidemic in 2020. It is important to consider that COVID-19 has since undergone multiple mutations, potentially leading to different characteristics compared to the time of our study. Second, the diagnoses of COVID-19 and non-COVID-19 pneumonia were based on the ICD-10 codes. The microbiological diagnosis of pneumonia was not available. Third, the database lacked laboratory data such as blood sampling results, imaging, and oxygen saturation. Information on the use of immunosuppressive agents were also unavailable.

In conclusion, our findings indicate that COVID-19 poses a significant risk of in-hospital mortality and a decline in ADL among patients on dialysis during the initial three waves of the pandemic. This suggests that the specific characteristics of COVID-19, along with the frailty of patients on dialysis and the prolonged isolation measures, may have contributed to these outcomes. The prolonged isolation period due to COVID-19 and the challenges in implementing rehabilitation interventions may contribute to the decline in ADL, particularly among patients receiving maintenance dialysis. These factors should be considered when determining the appropriate duration of isolation for dialysis patients with COVID-19.

### Supplementary Information

Supplementary Information.

## Data Availability

The data that support the findings of this study are available from the corresponding author S.M. upon reasonable request.

## References

[1] Bellan M (2021). Respiratory and psychophysical sequelae among patients with COVID-19 four months after hospital discharge. JAMA Netw. Open.

[2] Demeco A (2020). Rehabilitation of patients post-COVID-19 infection: A literature review. J. Int. Med. Res..

[3] Xu S (2021). Impact of the COVID-19 pandemic on health care utilization in a large integrated health care system: Retrospective cohort study. J. Med. Internet Res..

[4] Chakraborty I, Maity P (2020). COVID-19 outbreak: Migration, effects on society, global environment and prevention. Sci. Total Environ..

[5] Kalu IC, Henderson DK, Weber DJ, Haessler S (2023). Back to the future: Redefining ‘universal precautions’ to include masking for all patient encounters. Infect. Control Hosp. Epidemiol..

[6] de Oliveira Almeida K (2023). A systematic review on physical function, activities of daily living and health-related quality of life in COVID-19 survivors. Chronic Illness.

[7] Schoolwerth AC (2006). Chronic kidney disease: A public health problem that needs a public health action plan. Prev. Chronic Dis..

[8] Levey AS (2007). Chronic kidney disease as a global public health problem: Approaches and initiatives—A position statement from kidney disease improving global outcomes. Kidney Int..

[9] Tonelli M (2015). Comorbidity as a driver of adverse outcomes in people with chronic kidney disease. Kidney Int..

[10] Stevens LA (2010). Prevalence of CKD and comorbid illness in elderly patients in the United States: Results from the Kidney Early Evaluation Program (KEEP). Am. J. Kidney Dis..

[11] Collins AJ (2003). Chronic kidney disease and cardiovascular disease in the Medicare population. Kidney Int. Suppl..

[12] Avin KG, Moorthi RN (2015). Bone is not alone: The effects of skeletal muscle dysfunction in chronic kidney disease. Curr. Osteoporos. Rep..

[13] Bao Y, Dalrymple L, Chertow GM, Kaysen GA, Johansen KL (2012). Frailty, dialysis initiation, and mortality in end-stage renal disease. Arch. Intern. Med..

[14] Viasus D (2011). Epidemiology, clinical features and outcomes of pneumonia in patients with chronic kidney disease. Nephrol. Dial. Transplant..

[15] Ng JH (2020). Outcomes of patients with end-stage kidney disease hospitalized with COVID-19. Kidney Int..

[16] Mandai S (2017). Dialysis case volume associated with in-hospital mortality in maintenance dialysis patients. Kidney Int. Rep..

[17] Gr, B. International statistical classification of diseases and related health problems. Tenth revision. In *World Health Statistics Quarterly. Rapport trimestriel de statistiques sanitaires mondiales,* Vol. 41 (1988). 3376487

[18] Charlson ME, Pompei P, Ales KL, MacKenzie CR (1987). A new method of classifying prognostic comorbidity in longitudinal studies: Development and validation. J. Chronic Dis..

[19] Quan H (2011). Updating and validating the Charlson Comorbidity Index and score for risk adjustment in hospital discharge abstracts using data from 6 countries. Am. J. Epidemiol..

[20] Mandai S, Ando F, Mori T, Susa K, Iimori S, Naito S, Sohara E, Uchida S, Fushimi K, Rai T (2021). Burden of kidney disease on the discrepancy between reasons for hospital admission and death: An observational cohort study. PLoS ONE.

[21] Mahoney FI, Barthel DW (1965). Functional evaluation: The Barthel Index. Md. State Med. J..

[22] Shah S, Vanclay F, Cooper B (1989). Improving the sensitivity of the Barthel Index for stroke rehabilitation. J. Clin. Epidemiol..

[23] Hemmelgarn BR, Manns BJ, Quan H, Ghali WA (2003). Adapting the Charlson Comorbidity Index for use in patients with ESRD. Am. J. Kidney Dis..

[24] Kessler M, Frimat L, Panescu V, Briançon S (2003). Impact of nephrology referral on early and midterm outcomes in ESRD: EPidémiologie de l’Insuffisance REnale chronique terminale en Lorraine (EPIREL): Results of a 2-year, prospective, community-based study. Am. J. Kidney Dis..

[25] Kikuchi K (2021). Survival and predictive factors in dialysis patients with COVID-19 in Japan: A nationwide cohort study. Renal Replace. Ther..

[26] James MT (2009). CKD and risk of hospitalization and death with pneumonia. Am. J. Kidney Dis..

[27] Janus N, Vacher L-V, Karie S, Ledneva E, Deray G (2008). Vaccination and chronic kidney disease. Nephrol. Dial. Transplant..

[28] Mandai S (2021). Burden of kidney disease on the discrepancy between reasons for hospital admission and death: An observational cohort study. PLoS ONE.

[29] Chen H (2020). Declined functional status prolonged hospital stay for community-acquired pneumonia in seniors. Clin. Interv. Aging.

[30] Ali AM, Kunugi H (2021). Skeletal muscle damage in COVID-19: A call for action. Medicina.

[31] Welch C, Greig C, Masud T, Wilson D, Jackson TA (2020). COVID-19 and acute sarcopenia. Aging Dis..

[32] Fajgenbaum DC, June CH (2020). Cytokine storm. N. Engl. J. Med..

