# Supplementary material for: Impact of COVID-19 versus other pneumonia on in-hospital mortality and functional decline among Japanese dialysis patients: a retrospective cohort study
Source: Sci Rep. 2024 Mar 2;14:5177. doi: 10.1038/s41598-024-55697-z (PMC10908858; doi:10.1038/s41598-024-55697-z)
Supplement: Supplementary file 1 — Supplementary Information. [file 41598_2024_55697_MOESM1_ESM.pdf]

Impact of COVID-19 versus other pneumonia on in-hospital mortality and functional decline among Japanese dialysis patients: A retrospective cohort study

Ken Ikenouchi[1,2], Daiei Takahashi[2], Shintaro Mandai[1*], Mizuki Watada[2], Sayumi Koyama[2], Motoki Hoshino[2], Naohiro Takahashi[2], Wakana Shoda[2], Tamaki Kuyama[2], Yutaro Mori[1], Fumiaki Ando[1], Koichiro Susa[1], Takayasu Mori[1], Soichiro Iimori[1], Shotaro Naito[1], Eisei Sohara[1], Kiyohide Fushimi[3], and Shinichi Uchida[1*]

[1]Department of Nephrology, Graduate School of Medical and Dental Sciences, Tokyo Medical and Dental University, 1-5-45 Yushima, Bunkyo, Tokyo 113-8519, Japan
[2]Department of Nephrology, Musashino Red Cross Hospital, 1-26-1, Kyonann-cho, Musashino-shi, Tokyo, 180-8610, Japan
[3]Department of Health Policy and Informatics, Graduate School of Medical and Dental Sciences, Tokyo Medical and Dental University, 1-5-45 Yushima, Bunkyo, Tokyo 113-8519, Japan

Ikenouchi K. and Takahashi D. equally contributed to this study.

***Corresponding Authors**:
Shintaro Mandai and Shinichi Uchida
Department of Nephrology, Graduate School of Medical and Dental Sciences, Tokyo Medical and Dental University, 1-5-45 Yushima, Bunkyo, Tokyo 113-8519, Japan
Tel: +81-3-5803-5214; Fax: +81-3-5803-5215; E-mail: smandai.kid@tmd.ac.jp and suchida.kid@tmd.ac.jp

Figure S1. Patient flowchart.

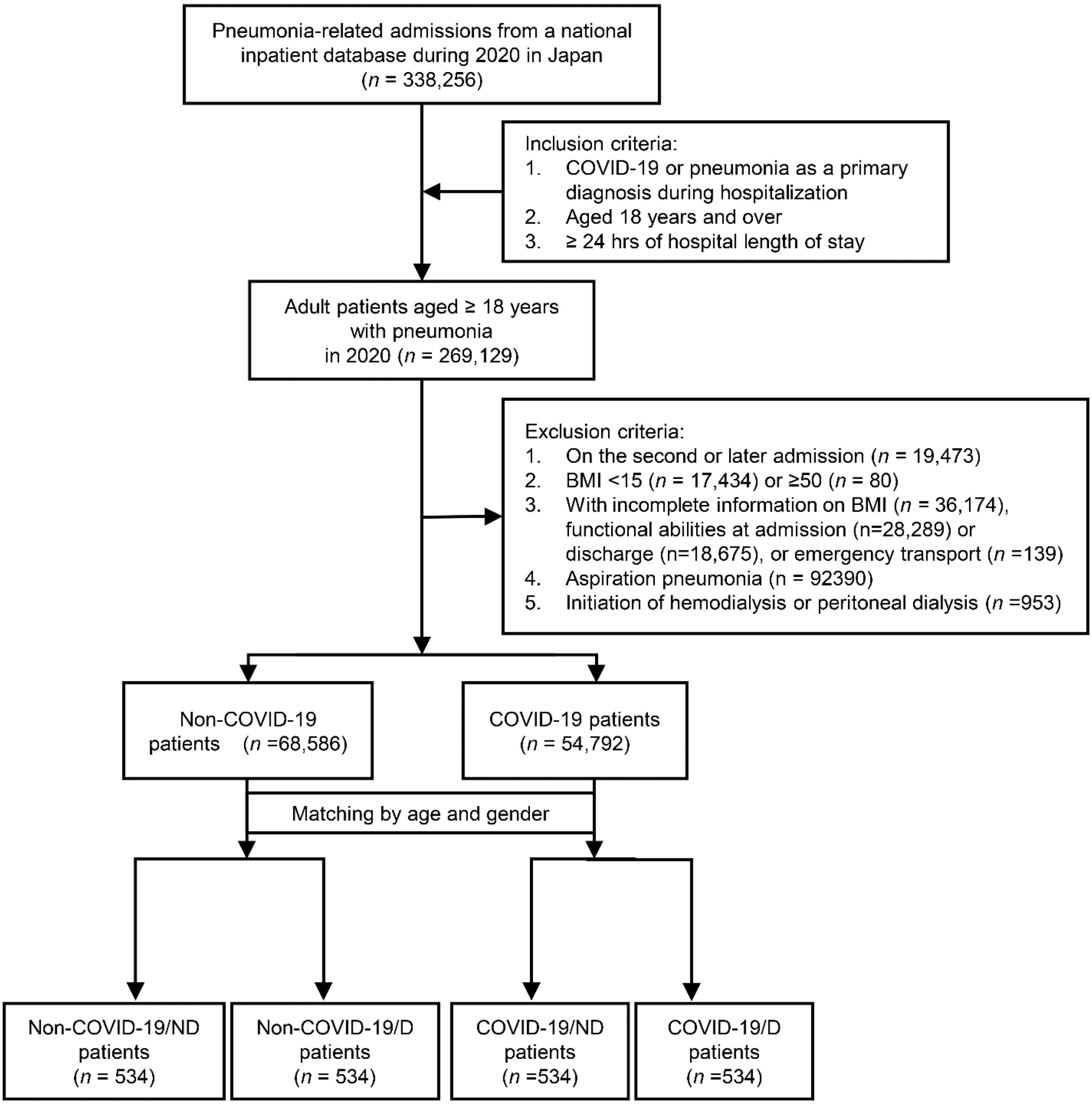

BMI, body mass index; ND, non-dialysis patients; D, dialysis; non-COVID-19, non-COVID-19 pneumonia

Figure S2. Risk of in-hospital death and decline in physical function among non-coronavirus disease 2019 (COVID-19)/non-dialysis, COVID-19/non-dialysis, non-COVID-19/dialysis, and COVID-19/dialysis patients, adjusting for diabetes mellitus.

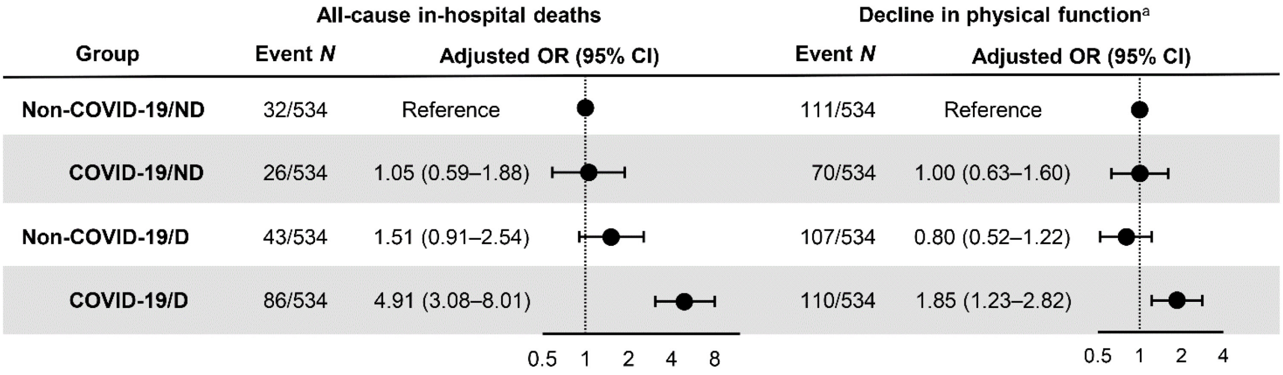

	All-cause in-hospital deaths		Decline in physical function[a]	
Group	Event *N*	Adjusted OR (95% CI)	Event *N*	Adjusted OR (95% CI)
Non-COVID-19/ND	32/534	Reference	111/534	Reference
COVID-19/ND	26/534	1.05 (0.59–1.88)	70/534	1.00 (0.63–1.60)
Non-COVID-19/D	43/534	1.51 (0.91–2.54)	107/534	0.80 (0.52–1.22)
COVID-19/D	86/534	4.91 (3.08–8.01)	110/534	1.85 (1.23–2.82)

Multivariable logistic regression models were adjusted for age, gender, body mass index, Barthel Index score on admission, and diabetes mellitus. Each circle represents a point estimate of odds ratios (ORs), and the solid lines represent the corresponding 95% confidence intervals (CIs).

[a] The decline in physical function was defined as a 20% or more decline in Barthel Index scores at discharge from admission.

CI, confidence interval; OR, odds ratio; ND, non-dialysis patients; D, dialysis; non-COVID-19, non-COVID-19 pneumonia

Figure S3. Risk of in-hospital death within 20 days or ≥21 days after admission among non-coronavirus disease 2019 (COVID-19)/non-dialysis, COVID-19/non-dialysis, non-COVID-19/dialysis, and COVID-19/dialysis patients.

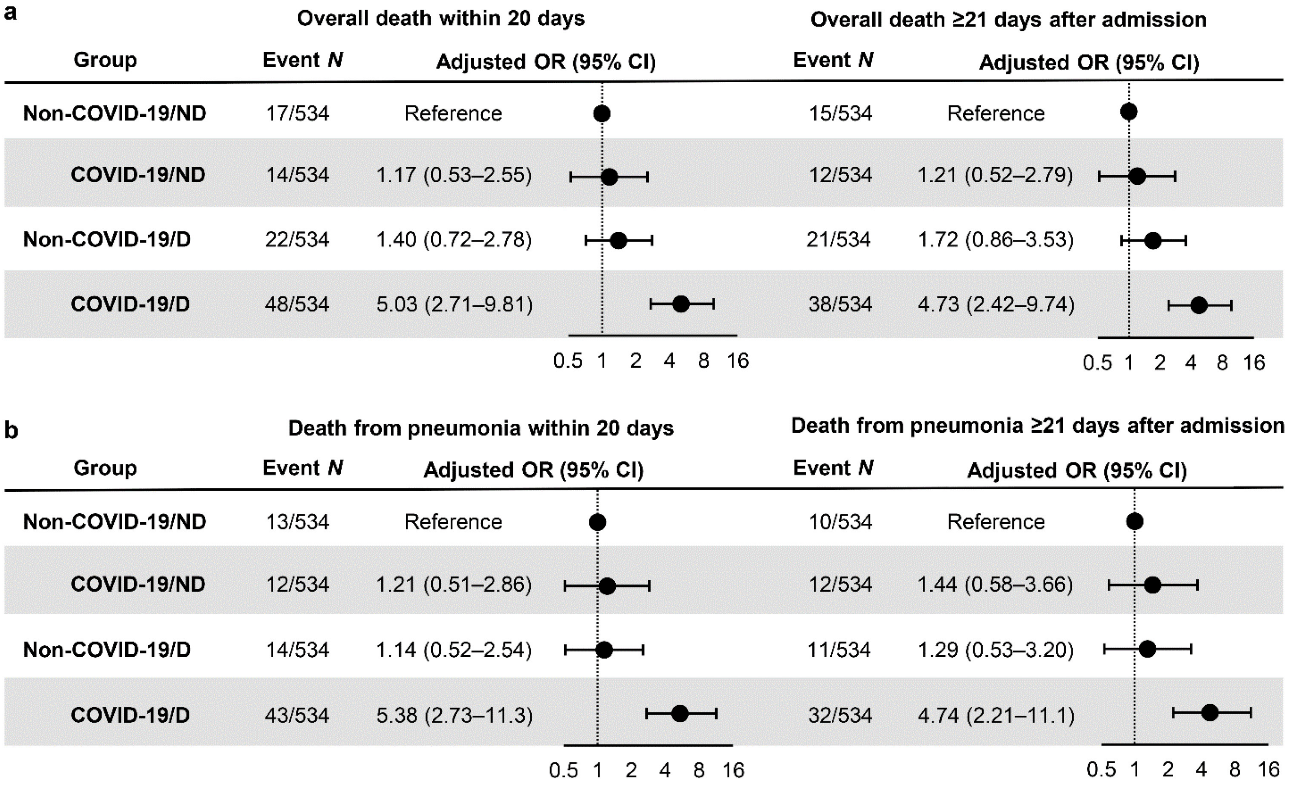

(a, b) Risks of in-hospital overall death (a) and death directly due to pneumonia (b) within 20 days or ≥21 days after admission among COVID-19/non-dialysis, COVID-19/non-dialysis, non-COVID-19/dialysis, and COVID-19/dialysis patients. Multivariable logistic regression models were adjusted for age, gender, body mass index, Barthel Index score on admission, and Charlson Comorbidity Index score. Each circle represents a point estimate of odds ratios (ORs), and the solid lines represent the corresponding 95% confidence intervals (CIs).

CI, confidence interval; OR, odds ratio; ND, non-dialysis patients; D, dialysis; non-COVID-19, non-COVID-19 pneumonia

Figure S4. Risk of longer hospital stays and high medical costs among non-coronavirus disease 2019 (COVID-19)/non-dialysis, COVID-19/non-dialysis, non-COVID-19/dialysis, and COVID-19/dialysis patients.

	Long-term hospitalization[a]		High medical cost[b]	
**Group**	**Event *N***	**Adjusted OR (95% CI)**	**Event *N***	**Adjusted OR (95% CI)**
**Non-COVID-19/ND**	67/534	Reference	51/534	Reference
**COVID-19/ND**	30/534	0.51 (0.32–0.82)	111/534	3.24 (2.19–4.79)
**Non-COVID-19/D**	85/534	1.37 (0.96–1.96)	98/534	2.15 (1.48–3.13)
**COVID-19/D**	84/534	1.61 (1.11–2.34)	274/534	13.2 (9.18-19.1)

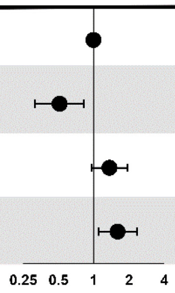

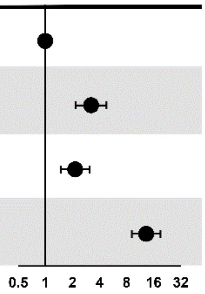

Multivariable logistic regression models were adjusted for age, gender, body mass index, Barthel Index score on admission, and Charlson Comorbidity Index score. Each circle represents a point estimate of odds ratios (ORs), and the solid lines represent the corresponding 95% confidence intervals (CIs).

[a] The long-term hospitalization was defined as a stay of 30 days or longer.

[b] The high medical cost was defined as the highest quartile of participants.

CI, confidence interval; OR, odds ratio; ND, non-dialysis patients; D, dialysis; non-COVID-19, non-COVID-19 pneumonia

Table S1. Characteristics of pre-match patients hospitalized with pneumonia during 2020

Variable	Non-COVID-19/ND (n = 66692)	COVID-19/ND (n = 54132)	Non-COVID-19/D (n = 1894)	COVID-19/D (n = 660)	P-value
Age, year	80 (72–87)	57 (41–72)	75 (68–82)	68 (57–77)	<0.0001
18–49	3646 (2.64)	20173 (22.83)	76 (2.35)	84 (8.25)	<0.0001
50–59	2928 (3.11)	9065 (16.20)	167 (6.61)	122 (15.16)	
60–69	6994 (8.83)	8303(17.62)	335 (15.81)	148 (21.87)	
70–79	17681 (25.60)	9141 (22.28)	705 (37.80)	184 (31.15)	
≥80	35443 (59.81)	7450 (21.07)	611 (37.43)	122 (23.57)	
Female	23813 (35.71)	22735 (42.00)	411 (21.70)	162 (24.55)	<0.0001
Body mass index, kg/m^2	20.83 (18.39–23.58)	23.46 (20.96–26.44)	20.73 (18.47–23.52)	22.95 (20.38–25.81)	<0.0001
Barthel Index score					
0–20	23017 (34.51)	5105 (9.43)	558 (29.46)	103 (15.61)	<0.0001
21–60	11762 (17.64)	2713 (5.01)	383 (20.22)	85 (12.88)	
61–90	6956 (10.43)	2174 (4.02)	237 (12.51)	56 (8.48)	
91–100	24957 (37.42)	44140 (81.54)	716 (37.80)	416 (63.03)	
Charlson Comorbidity Index					
Myocardial infarction	1303 (1.95)	502 (0.93)	63 (3.33)	8 (1.21)	<0.0001
Congestive heart failure	14614 (21.91)	1272 (2.35)	638 (33.69)	42 (6.36)	<0.0001
Peripheral vascular disease	1106 (1.66)	180 (0.33)	105 (5.54)	7 (1.06)	<0.0001
Cerebrovascular disease	7182 (10.77)	1070 (1.98)	197 (10.40)	28 (4.24)	<0.0001
Dementia	8312 (12.46)	1195 (2.21)	132 (6.97)	14 (2.12)	<0.0001
Chronic pulmonary disease	13029 (19.54)	2711 (5.01)	114 (6.02)	20 (3.03)	<0.0001
Rheumatologic disease	2624 (3.93)	463 (0.86)	24 (1.27)	4 (0.61)	<0.0001
Peptic ulcer disease	1821 (2.73)	819 (1.51)	43 (2.27)	16 (2.42)	<0.0001
Mild liver disease	1983 (2.97)	1135 (2.10)	48 (2.53)	6 (0.91)	<0.0001
Diabetes without chronic complications	11226 (16.83)	6874 (12.70)	261 (13.78)	66 (10.00)	<0.0001
Diabetes with chronic complications	2160 (3.24)	764 (1.41)	475 (25.08)	133 (20.15)	<0.0001
Hemiplegia or paraplegia	129 (0.19)	24 (0.04)	4 (0.21)	1 (0.15)	<0.0001
Any malignancy, including leukemia and lymphoma	9391 (14.08)	1305 (2.41)	154 (8.13)	13 (1.97)	<0.0001
Moderate or severe liver disease	103 (0.15)	31 (0.06)	2 (0.11)	2 (0.30)	<0.0001

Metastatic solid tumor	1520 (2.28)	133 (0.25)	8 (0.42)	0 (0.00)	<0.0001
AIDS/HIV	102 (0.15)	106 (0.20)	0 (0.00)	1 (0.15)	<0.0001
Charlson comorbidity index					
0	24776 (37.15)	45827 (84.66)	51 (2.69)	182 (27.58)	<0.0001
1–2	28346 (42.50)	6955 (12.85)	997 (52.64)	404 (61.21)	
≧3	13570 (20.35)	1350 (2.49)	846 (44.67)	74 (11.21)	

Data are presented as numbers (percentages) or medians (interquartile ranges). To compare the four groups, one-way analysis of variance was used for continuous variables, while a chi-squared test was used for categorical variables.

ND, non-dialysis; D, dialysis; non-COVID-19, non-COVID-19 pneumonia
